# A Home to Die in

**Published:** 1889-11-16

**Authors:** 


### A Home to Die In.

"Je meurrai seule" it is the cry of many a sad and anxious heart. When youth and happiness are present the fear of death is far away, but when sickness comes, when friends die and leave us, that terrible weight of foreboding falls heavily on the troubled mind, and finds expression in the words, "I shall die alone." Why, we ask, in the animal world is a solitary death unfeared, while in the world of men it is more dreaded than any other possible catastrophe? It is not difficult to explain. Sympathy, kindness, the loving look of understanding, reach the soul. In our modern life sympathy plays so large a part that it is useless for us to reason on the possibilities of a more stoical form of thought, and it is only the rare and independent character whom we occasionally find to half wonder at, half admire, who, like the wounded animal, longs to retire within himself and end the game of life by himself. Sister Dora, we are told, died alone. She had loving friends in the next room, but they were, by her special request, not present at her death. "I have lived alone, let me die alone," said this strangely proud and independent-spirited woman.

But it is only to the few that such independence of human sympathy is given. Who could think the many hundreds of sick and suffering that we know are brave enough to face death by themselves? Sister Dora died of a long and painful illness; it is of the sufferers from that most dreaded disease—cancer—that I wish to write more especially. The word itself brings horror to many a mind, yet can they not bear to think of it for a moment, to realise what it means to those lonely ones who are doomed to suffer perhaps for years, who must endure the long misery of hopeless pain with, alas, too often the added anxiety of a lonely death or one in the workhouse wards. There is, I suppose, no greater trial than this most cruel disease. Physicians and surgeons do their utmost, and yet hundreds die yearly from it.

Let us soften these lives, let us make the pain less, we more fortunate ones who can tend our loved ones with tender care; let us feel for those often lonely, often old or middle-aged sufferers who are dying of this terrible disease. One noble hospital in London reserves a certain number of beds for those patients who are afflicted with cancer. Here they are given every chance of care; if the cases are hopeless they are kept and cared for to the end. In the sunny wards of the Middlesex Hospital since the year 1792 patients suffering from cancer have been nursed and cared for till "relieved by art or released by death."

This charity was founded by Mr. Samuel Whitbread, whose munificence was, at his earnest desire, kept secret, till after death his name was revealed. It is unique throughout the world. Here the victims of this almost hopeless disease find alleviation for their pain. As the hospital report says: "No one who has interested himself in the unhappy lot of these patients, usually exceeding thirty-five in number, can have failed to observe how greatly their mental suffering is mitigated by the knowledge that they have here offered to them, if they so desire it, a peaceful and permanent refuge for the remainder of their existence. In it they are exempted from the anxious cares of providing for the daily wants of life, as well as from the sad necessity of repeated solicitations for another and another refuge, as the term for which they can be received into other hospitals from time to time approaches to its close."

One certainly cannot help observing the loving care that surrounds these patients. On the day on which I went over the wards I saw the eyes of many brighten as the matron approached them with a kind word or suggestion for their comfort. Game had been just sent to the hospital, and the poor patients of the cancer ward were to share the pleasure afforded by a change of food. One old man, who seemed in great pain, and who had eaten little for some days, brightened up at the idea of a partridge, and remarked that he "had been always very partial to game." One cannot doubt there would be no lack of presents of this kind to the hospital if the donors knew what a help such gifts are in tempting failing appetites. One charming addition has lately been made to the male cancer wards—namely, a smoking-room, and a most pleasant room it is, full of sunshine on the day we visited it, prettily furnished and with a comfortable settle facing the large bow-window. Nothing more ideal for those patients who can leave their beds could be imagined. The men here expressed themseves as "feeling a little easier." One cannot fail to feel that when the worst attacks of pain are absent such bright pretty surroundings are very helpful in alleviating the depression which is one of the most painful results of this sad disease. "How is it supported?" we asked, before leaving. "Well," said the matron, "the female wards are endowed, but, alas! we always want money for them. These poor things have exactly what they 'fancy' in the way of food, and this costs a good deal. Then only eight beds in the male wards are endowed, and this is a source of anxiety to us. This ward has been a recent addition, as the first endowment only included female patients, but the male wards were almost a necessity." Naturally, I thought, for a sick man is even a more sad and helpless sight than a sick woman. Here in that sunny window, overlooking the hospital garden, these poor men can sit and smoke their pipe (one of the few enjoyments left them), and they can watch the trees grow green and brown with the varying seasons. The room is warm and bright, birds are singing, flowers are about, and there is the unmistakable atmosphere of care and love around. Will not the generous public, in memory of the many dear ones who are gone and whose pain is over, help this effort of love for those who dwell still in this "house of suffering," and who must wait in it till "art relieves or death releases them"? Surely these male cancer wards of the Middlesex Hospital will soon have no one bed left unendowed.

The charity is a great and noble one, and it seems a privilege to help in carrying it out in the most perfect manner.

